# Advanced Hemodynamic Management in Patients with Septic Shock

**DOI:** 10.1155/2016/8268569

**Published:** 2016-09-14

**Authors:** Bernd Saugel, Wolfgang Huber, Axel Nierhaus, Stefan Kluge, Daniel A. Reuter, Julia Y. Wagner

**Affiliations:** ^1^Department of Anesthesiology, Center of Anesthesiology and Intensive Care Medicine, University Medical Center Hamburg-Eppendorf, Martinistrasse 52, 20246 Hamburg, Germany; ^2^II. Medizinische Klinik und Poliklinik, Klinikum rechts der Isar der Technischen Universität München, Ismaninger Strasse 22, 81675 München, Germany; ^3^Department of Intensive Care Medicine, Center of Anesthesiology and Intensive Care Medicine, University Medical Center Hamburg-Eppendorf, Martinistrasse 52, 20246 Hamburg, Germany

## Abstract

In patients with sepsis and septic shock, the hemodynamic management in both early and later phases of these “organ dysfunction syndromes” is a key therapeutic component. It needs, however, to be differentiated between “early goal-directed therapy” (EGDT) as proposed for the first 6 hours of emergency department treatment by Rivers et al. in 2001 and “hemodynamic management” using advanced hemodynamic monitoring in the intensive care unit (ICU). Recent large trials demonstrated that nowadays protocolized EGDT does not seem to be superior to “usual care” in terms of a reduction in mortality in emergency department patients with early identified septic shock who promptly receive antibiotic therapy and fluid resuscitation. “Hemodynamic management” comprises (a) making the diagnosis of septic shock as one differential diagnosis of circulatory shock, (b) assessing the hemodynamic status including the identification of therapeutic conflicts, and (c) guiding therapeutic interventions. We propose two algorithms for hemodynamic management using transpulmonary thermodilution-derived variables aiming to optimize the cardiocirculatory and pulmonary status in adult ICU patients with septic shock. The complexity and heterogeneity of patients with septic shock implies that individualized approaches for hemodynamic management are mandatory. Defining individual hemodynamic target values for patients with septic shock in different phases of the disease must be the focus of future studies.

## 1. Introduction

A recent consensus report defines sepsis as “life-threatening organ dysfunction caused by a dysregulated host response to infection” [1]. Septic shock is defined as a “subset of sepsis in which particularly profound circulatory, cellular, and metabolic abnormalities are associated with a greater risk of mortality than with sepsis alone” [1]. Clinical indicators of septic shock are defined by a need for vasopressor administration to maintain a mean arterial pressure (MAP) of 65 mmHg or greater and a serum lactate level greater than 2 mmol/L in the absence of hypovolemia [1].

Complex disease syndromes such as septic shock require multimodal diagnostic and therapeutic approaches. Besides the diagnosis of septic shock and early causal therapy one major challenge in its treatment remains the resuscitation and management of cardiocirculatory and respiratory dysfunction.

In this context, the hemodynamic management in both early and later phases of these syndromes is crucial. However, with regard to the management of cardiovascular dynamics in patients with sepsis and septic shock we still have more questions than answers.

In this article, we therefore aim to expand on the difference between “early goal-directed therapy” (EGDT) and “hemodynamic management” and propose an approach for goal-directed hemodynamic management in patients with septic shock.

## 2. Early Goal-Directed Therapy in Septic Shock

In 2001, Rivers et al. published their monocentric randomized controlled landmark study describing that EGDT during the first 6 hours of treatment markedly reduced mortality among patients admitted to the emergency department with severe sepsis or septic shock [2]. The 6-hour EGDT algorithm as proposed by Rivers et al. is a multimodal protocolized therapeutic approach targeting a central venous pressure (CVP) of 8–12 mmHg (by giving fluids), a MAP of 65–90 mmHg (by giving vasoactive agents), and a central venous oxygen saturation (ScvO_2_) of ≥70% (by transfusion of red blood cells or administration of inotropic agents) [2]. Of note, in both the study and the control group, more than 94% of patients received immediate adequate antibiotic therapy [2]. This study essentially contributed to the notion that “optimization” of hemodynamics during the first hours of treatment can markedly lower mortality of patients with sepsis. The Surviving Sepsis Campaign (SSC) guidelines adopted those treatment goals in the recommendations for the “initial resuscitation” within the “6-hour bundles” [3].

The concept of EGDT as proposed by Rivers et al. has recently been questioned by three large multicenter randomized controlled trials—the ProCESS [4], ARISE [5], and PROMISE trial [6]—and an updated meta-analysis [7]. These trials demonstrated that nowadays protocolized EGDT for the first 6 hours (including monitoring of ScvO_2_ and liberal red blood cell transfusion) seems not to be superior to “usual care” in terms of a reduction in mortality in emergency department patients with septic shock. It needs to be stressed, however, that, in all three trials, patients were identified early as having septic shock (in contrast to the Rivers trial) and promptly received antibiotic therapy and fluid resuscitation. In addition, the mortality observed in the control groups was markedly lower in the current trials compared with the Rivers study. Therefore, one can conclude that “usual resuscitation” has improved since the Rivers study and that the SSC guidelines increased the awareness for sepsis and its early recognition and its treatment with antibiotics and intravenous fluid [6, 8].

In addition, baseline characteristics of the patients of the three recent trials differed from those of the Rivers study with regard to severity of illness and timing of diagnosis of septic shock (lower lactate levels, higher ScvO_2_, and lower APACHE II score) [9, 10]. Thus, the results of these trials might lack “external validity.” In particular, the question about potentially beneficial effects of goal-directed therapy in patients with very severe septic shock, in patients who do not respond to initial therapy, or in patients in whom the diagnosis of septic shock is established at a later point in time cannot be answered by these trials [9].

Nevertheless, in response to this new evidence, the SSC recently changed their bundle recommendations with regard to hemodynamic resuscitation within the first 6 hours [11]. Instead of targeting distinct values of CVP and ScvO_2_ the guidelines now recommend to “re-assess volume status and tissue perfusion” by repeated “focused exam (…) including vital signs, cardiopulmonary, capillary refill, pulse, and skin findings” or “two of the following: measure CVP, measure ScvO_2_, bedside cardiovascular ultrasound, dynamic assessment of fluid responsiveness with passive leg raise or fluid challenge.” These updated recommendations reflect the recent evidence from the three large randomized controlled trials as well as the widely accepted importance of assessment of fluid responsiveness.

Key elements in the initial treatment of sepsis therefore remain the early recognition of sepsis and early antibiotic therapy and source control. Hemodynamic therapy should aim at the optimization of intravascular volume status, perfusion pressure, and blood flow to restore tissue perfusion. However, major questions regarding the optimization of cardiovascular dynamics remain as will be discussed in the next paragraphs.

## 3. The Difference between “Early Goal-Directed Therapy” and “Hemodynamic Management”

The concept of EGDT needs to be differentiated from “hemodynamic management” of patients with septic shock.

EGDT as described by Rivers et al. [2] and reassessed by the three large trials described above [4–6] only covers the first 6 hours of resuscitation of patients with sepsis and septic shock and is usually applied in emergency department patients presenting with suspected or confirmed sepsis or septic shock.

Therefore, EGDT is based on the basic hemodynamic variables CVP, MAP, and ScvO_2_. From a pathophysiologic point of view the use of these hemodynamic targets to guide therapy with fluids, vasopressors, and inotropes can be questioned [12]. CVP has limited capabilities to reflect intravascular volume status and fluid responsiveness [13–15] and its use as a resuscitation goal in EGDT might lead to fluid overload [16, 17]. With regard to MAP, individual target values are not well described [18]. ScvO_2_ is an unspecific parameter of the balance between oxygen delivery and oxygen consumption. It has been shown that ScvO_2_ is below 70% in only about 27% of septic shock patients in the first hours after admission to the intensive care unit (ICU) [19]. Moreover, even in patients with high ScvO_2_ a mismatch between oxygen delivery and consumption may be present.

Patients with septic shock regularly require intensive care for days or even weeks. “Hemodynamic management,” therefore, refers to the diagnostic and therapeutic approaches aiming to identify and resolve cardiocirculatory alterations during the complete course of septic shock—from initial differential diagnosis to early resuscitation and hemodynamic therapy of patients with septic shock associated with complex complications such as acute respiratory distress syndrome (ARDS), renal failure, abdominal compartment syndrome, or preexisting myocardial dysfunction.

“Hemodynamic management” can utilize advanced hemodynamic parameters (reflecting global blood flow, myocardial contractility, intravascular volume status, fluid responsiveness, and cardiac afterload) assessed with a variety of different techniques such as echocardiography, pulmonary artery catheterization and thermodilution, transpulmonary thermodilution, and calibrated and uncalibrated pulse contour analysis. In addition, functional tests (passive leg raising test [20] and fluid challenge test [21]) are used to assess fluid responsiveness, that is, an increase in cardiac output (CO) after administration of fluid.

## 4. Hemodynamic Management of Patients with Septic Shock

Hemodynamic management comprises (a) making the diagnosis of septic shock (as one differential diagnosis of circulatory shock), (b) assessing the hemodynamic status (volume status, fluid responsiveness, need for vasopressor, or inotropic agent) including the identification of therapeutic conflicts (e.g., intravascular hypovolemia in the presence of pulmonary fluid overload), and (c) guiding therapeutic interventions.

The hemodynamic management of septic shock patients remains a complex challenge. There are no SSC guideline recommendations on the hemodynamic management for the period following the initial 6 hours of treatment in septic shock [3]. A consensus conference report of the European Society of Intensive Care Medicine (ESICM) can provide guidance on how to perform hemodynamic monitoring in critically ill patients with circulatory shock [22].

### 4.1. Differential Diagnosis of Cardiovascular Pathophysiology and Diagnosis of Septic Shock

In patients with circulatory shock, the identification of the type of shock is crucial to adequately guiding causal and supportive therapeutic approaches [22, 23]. Signs of poor tissue perfusion indicative for the presence of circulatory shock can be found on physical examination [24, 25]. However, using physical examination for identifying the underlying type of shock and the specific hemodynamic alterations is challenging [26–29]. Therefore, if physical examination does not lead to a clear diagnosis of the underlying type of shock, further hemodynamic assessment by echocardiography or—in complex patients—advanced hemodynamic monitoring (pulmonary artery catheter or transpulmonary thermodilution) is recommended [22]. In patients with septic (i.e., distributive) shock, increased CO (hyperdynamic circulatory failure), normal or decreased intravascular fluid status, and markedly decreased systemic vascular resistance are characteristic findings. However, in patients with impaired myocardial contractility (e.g., because of ischemic or septic cardiomyopathy) or hypovolemia, CO can also be decreased.

### 4.2. Hemodynamic Monitoring for the Assessment of the Hemodynamic Status

The serial assessment of the hemodynamic status of a patient with septic shock is crucial to identifying the therapeutic options to optimize perfusion pressure and global blood flow in order to restore and optimize tissue perfusion. Since both hypovolemia and hypervolemia are associated with unfavorable outcomes [17, 30], assessment of the hemodynamic status (including volume status and fluid responsiveness) remains a key challenge in the treatment of septic shock. While CVP, cardiac filling pressures, and static volumetric parameters of cardiac preload alone should not be used to guide fluid therapy, fluid therapy based on more than one single hemodynamic variable and the use of dynamic parameters (pulse pressure variation and stroke volume variation that can only be used in patients with sinus rhythm and controlled mechanical ventilation) is recommended by the ESICM consensus report [22]. Because appropriate clinical hemodynamic endpoints to guide and titrate therapy with fluids are poorly defined, a careful titration of fluids especially in the presence of elevated filling pressures and extravascular lung water has been suggested [22]. To predict the patient's response to a fluid bolus, a passive leg raising test, that is, autotransfusion of blood from the lower extremities to the thoracic compartment, can be performed [31, 32]. The clinical gold standard test to evaluate fluid responsiveness is the actual administration of a fluid bolus and the continuous monitoring of CO to monitor the hemodynamic response during this fluid challenge test [21]. It has to be mentioned, however, that the concept of fluid responsiveness is based on pathophysiologic considerations and has not been rigorously evaluated in randomized controlled trials [33].

### 4.3. Hemodynamic Management in Septic Shock: Therapeutic Conflicts

If septic shock is complicated by ARDS, therapeutic conflicts between absolute or relative intravascular hypovolemia causing circulatory shock and pulmonary fluid overload remain key challenges in the hemodynamic management. Therefore, the ESICM expert consensus on circulatory shock and hemodynamic monitoring suggests the use of advanced hemodynamic monitoring in patients with severe shock (especially if complicated by ARDS) [22]. Pulmonary artery catheterization remains reasonable in septic shock patients with right ventricular failure or pulmonary artery hypertension [34]. However, the use of the pulmonary artery catheter has declined in the ICU setting during the recent years for a variety of reasons including its invasiveness and the availability of less-invasive hemodynamic monitoring technologies [35].

In septic shock accompanied by ARDS, hemodynamic management based on transpulmonary thermodilution can add additional valuable information about extravascular lung water index (EVLWI) [36] and pulmonary vascular permeability [37]. EVLWI gives useful prognostic information regarding mortality in critically ill patients in general, in patients with sepsis or septic shock, and in patients with ARDS [36]. Recent data demonstrate that EVLWI is of high prognostic value during fluid resuscitation in septic patients after initial resuscitation [38]. Because transpulmonary thermodilution allows the measurement of CO, volumetric cardiac preload parameters, and EVLWI and the calculation of systemic vascular resistance, its use might help to guide fluid therapy and therapy with vasopressors and inotropes in complex septic shock patients even in the context of pulmonary fluid overload. Transpulmonary thermodilution has therefore recently been suggested to be used in shock patients without ARDS not responding to initial therapy or in patients with shock and ARDS [39]. The combined application of transpulmonary thermodilution and calibrated pulse contour analysis additionally allows the continuous estimation of CO during functional diagnostic tests aiming to assess fluid responsiveness.

### 4.4. Hemodynamic Management: Different Phases of Resuscitation and “Deresuscitation”

Although it is generally agreed upon that fluid administration and vasopressor therapy are key components in the hemodynamic management of patients with septic shock, basic questions about the timing of these therapeutic interventions largely remain unanswered or controversial. Although the SSC guidelines recommend “aggressive fluid resuscitation during the first 24 hours of management” [3], dosing and timing of intravenous fluid administration remain largely empirical [40–42].

Hypovolemia is associated with tissue hypoperfusion and organ failure [43]. The rationale behind fluid therapy in hemodynamically compromised patients is to increase oxygen delivery by increasing stroke volume (and thus CO) [40]. This is based on the physiologic relation of cardiac preload and stroke volume as described by the Frank-Starling cardiac function curve [40]. Since fluid loading transiently increases the stressed blood volume and venous return (by increasing the gradient between mean systemic filling pressure and right atrial pressure) fluid administration can result in an increase of stroke volume in patients on the ascending part of the Frank-Starling curve [43–45].

Despite this sound physiologic concept, it is important to consider that only about 50% of critically ill patients are in a hemodynamic state of fluid responsiveness [40, 45]. In addition, increasing CO by fluid loading is probably only justifiable if signs of tissue hypoperfusion are present [40].

Aggressive fluid resuscitation has been found to be independently associated with worse outcomes in critically ill patients including organ dysfunction and mortality [30, 46]. Excessive fluid resuscitation results in tissue edema impairing endothelial integrity, microcirculatory blood flow, and diffusion of oxygen and metabolites and finally results in impaired organ blood flow [30, 45]. A negative fluid balance, conversely, has been shown to be associated with survival in patients with sepsis [47] and with improved pulmonary organ function in patients with ARDS [48].

In the absence of definite evidence regarding the optimal timing of fluid administration in septic shock, concepts for hemodynamic management taking into account different phases of fluid resuscitation and “deresuscitation” have been proposed. An early transition to a conservative fluid management or even “late goal-directed fluid removal” following the initial resuscitation phase characterized by the liberal administration of fluids has been suggested [45]. Marik even suggested a primarily conservative approach of fluid bolus administration (in contrast to the SSC recommendations) [46]. With regard to factors that have to be considered during fluid therapy, Malbrain et al. emphasized the importance of 4 key elements: drug, dosing, duration, and deescalation [49].

However, these concepts and suggestions further need to be evaluated in observational and interventional clinical studies before they can be recommended for routine clinical practice.

## 5. Advanced Hemodynamic Monitoring Using Transpulmonary Thermodilution in the Hemodynamic Management of Septic Shock

### 5.1. Transpulmonary Thermodilution

Transpulmonary thermodilution allows the determination of a variety of hemodynamic variables in patients equipped with a central venous catheter placed in the superior or inferior vena cava and a dedicated thermistor-tipped arterial catheter that is usually placed in the abdominal aorta through the femoral artery [50–53]. Single-indicator transpulmonary thermodilution techniques that are now commercially available from two different manufacturers [52, 54] have been developed based on the experience with double-indicator (thermodye) transpulmonary thermodilution [55]. When using single-indicator transpulmonary thermodilution, a thermal indicator (cooled saline) is injected in the central venous circulation and passes the right heart, the pulmonary circulation, and the left heart. Subsequently, the thermal indicator bolus is detected by the thermistor located at the tip of the arterial catheter and a curve reflecting the dilution of the cold indicator on its way through cardiopulmonary circulation is derived. Further analysis of this thermodilution curve allows the calculation of various hemodynamic parameters for the assessment of CO, myocardial contractility, cardiac preload, and EVLWI [52, 54, 56–60]. In short, CO is calculated from the thermodilution curve by applying a modified Stewart-Hamilton algorithm [61, 62]. Based on two further main parameters characterizing the thermodilution curve—that is, mean transit time (MTt) and the downslope time (DSt)—global end-diastolic volume index (GEDVI) and EVLWI can be computed as described in detail before [59, 63–68]. GEDVI can be used to estimate cardiac preload (volumetric cardiac preload parameter) and EVLWI that is elevated in patients with pulmonary edema or pneumonia is a marker of fluid outside of the pulmonary vasculature [59, 63–68].

### 5.2. Data on Transpulmonary Thermodilution in the Hemodynamic Management of Septic Shock Patients

Despite the pathophysiologic rationale for advanced hemodynamic management in septic shock patients and the expert consensus recommendations for its use described above [22], to date, definite algorithms to guide fluid therapy using advanced hemodynamic monitoring with transpulmonary thermodilution cannot be generally recommended based on the existing literature.

In general, hemodynamic monitoring per se will never influence patient outcome unless measured hemodynamic variables trigger meaningful and reasonable therapeutic interventions that are able to improve outcome [69].

In a two-center, randomized trial in septic and nonseptic shock patients, Trof et al. compared two hemodynamic management algorithms using predefined values of different hemodynamic parameters as upper resuscitation limits—transpulmonary thermodilution-derived values of EVLWI and GEDVI in one group and pulmonary artery occlusion pressure in the other group [70]. The authors observed no clinically relevant or statistically significant difference in the primary endpoints (ventilator-free days and ICU and hospital length of stay), organ failure, and mortality. Therapy guided by GEDVI and EVLWI resulted in a more positive fluid balance. However, the study protocol used by the authors was repeatedly criticized for the endpoints chosen to serve as an upper limit to administer fluid [71–74].

Another example that illustrates the problem of questionable treatment algorithms is a prospective trial by Zhang et al. comparing hemodynamic treatment based on transpulmonary thermodilution-derived variables with CVP-based management in patients with septic shock with or without ARDS. The trial was stopped prematurely and did not show any statistically significant differences in the primary endpoint (28-day mortality) and secondary endpoints [75]. Again, major flaws in the study design and the hemodynamic treatment protocol might explain the authors' findings [76–78]; for example, applying the same hemodynamic algorithm to septic shock patients with and without ARDS seems to be counterintuitive and against basic pathophysiologic principles [76, 78]. In addition, questionable therapeutic interventions (low-molecular starch 130/0.4 and diuretics) were applied triggered by questionable cut-off values of transpulmonary thermodilution-derived variables [76].

In another study, a hemodynamic treatment algorithm to guide fluid administration in patients with septic shock was described which is based on functional cardiac preload parameters (pulse pressure variation if applicable or changes in stroke volume after a passive leg raising test) and transpulmonary thermodilution-derived cardiac index (CI) [79]. There was no difference in the time till resolution of shock (primary endpoint) between the study group and the control group in which fluid administration was guided by an algorithm primarily based on CVP. In the study group, however, the amount of fluids administered per day was lower compared to the control group.

Considering the results of these studies, the conclusion that advanced hemodynamic management is useless to improve outcome in septic patients is not justified. Those studies rather suggest that further trials investigating hemodynamic treatment strategies in septic shock patients are necessary. It must be the aim to develop treatment algorithms aiming at a rational optimization of appropriate pathophysiologically reasonable hemodynamic target variables. These algorithms then need to be thoroughly evaluated in randomized controlled trials in clearly characterized patient collectives using reasonable outcome measures.

### 5.3. Transpulmonary Thermodilution in the Hemodynamic Management of Septic Shock: Suggestion for a Treatment Algorithm

**Figure 1:**
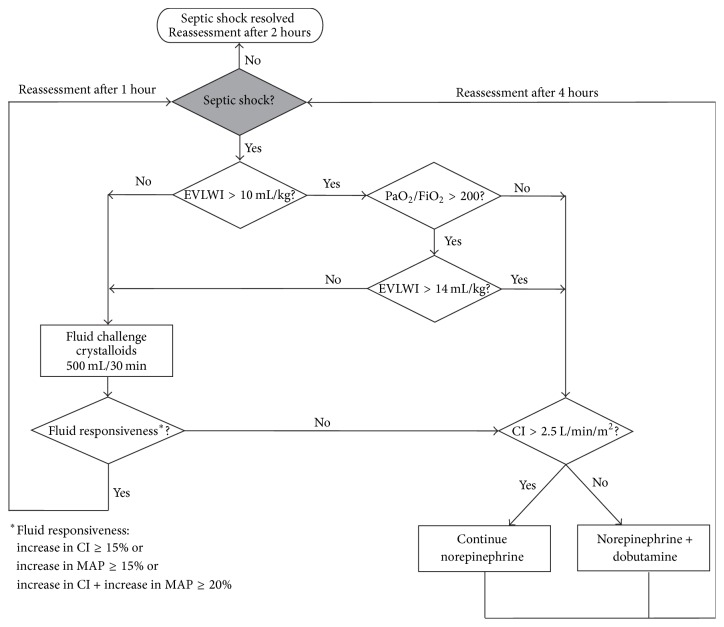
Algorithm  1—treatment algorithm for the first 24 hours of hemodynamic management. Hemodynamic therapeutic interventions in Algorithm  1 include fluids (crystalloids), a vasopressor (norepinephrine), and an inotrope (dobutamine) titrated according to extravascular lung water index (EVLWI) and cardiac index (CI). Crystalloids are given as a bolus of 500 mL (fluid challenge). We define fluid responsiveness as an increase in CI of ≥15% or an increase in mean arterial pressure (MAP) of ≥15% or a cumulative increase in CI and MAP of ≥20% (given that the dose of vasopressors is kept constant). EVLWI is complemented by the arterial partial pressure of oxygen (PaO_2_)/fraction of inspired oxygen (FiO_2_) ratio to account for the individual pulmonary function of the patient.

**Figure 2:**
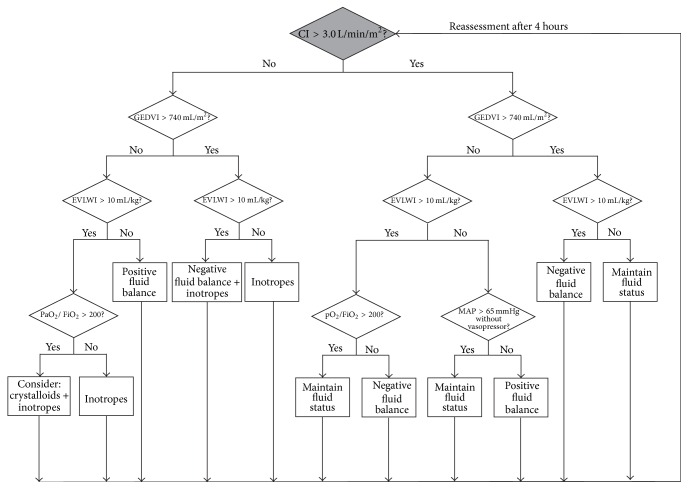
Algorithm  2—treatment algorithm for hemodynamic management during the intensive care unit stay following the initial 24 hours. Algorithm  2 gives treatment recommendations based on cardiac index (CI), global end-diastolic volume index (GEDVI), extravascular lung water index (EVLWI), and mean arterial pressure (MAP). EVLWI is complemented by the arterial partial pressure of oxygen (PaO_2_)/fraction of inspired oxygen (FiO_2_) ratio to account for the individual pulmonary function of the patient. During treatment according to Algorithm  2 all patients receive norepinephrine to maintain a MAP of ≥65 mmHg.

In the following we propose two hemodynamic treatment algorithms using transpulmonary thermodilution-derived variables aiming to optimize the cardiocirculatory and pulmonary status in adult ICU patients with septic shock as defined by the recent consensus definition [1]. An outline of the algorithms is given in Figures 1 and 2. These algorithms are based on pathophysiologic rationale, clinical experience, and available data from previous studies. The algorithms have not been tested in a randomized controlled trial and—in the absence of definite evidence on this topic—are meant to initiate a discussion on how advanced hemodynamic monitoring using transpulmonary thermodilution might be performed in an algorithmic approach in septic shock.

According to the different phases of hemodynamic resuscitation described above we distinguish between an algorithm for the early phase, that is, during the first 24 hours of treatment (Algorithm  1) and an algorithm for further hemodynamic management (Algorithm  2).

Hemodynamic therapeutic interventions in Algorithm  1 include fluids (crystalloids), a vasopressor (norepinephrine), and an inotrope (dobutamine) titrated according to EVLWI and CI. Crystalloids are given as a bolus of 500 mL (fluid challenge) [46]. We define fluid responsiveness as an increase in CI of ≥15% or an increase in MAP of ≥15% or a cumulative increase in CI and MAP of ≥20% (given that the dose of vasopressors is kept constant). EVLWI is complemented by the arterial partial pressure of oxygen (PaO_2_)/fraction of inspired oxygen (FiO_2_) ratio to account for the individual pulmonary function of the patient.

Algorithm  2 gives more general treatment recommendations based on CI, GEDVI, EVLWI (again complemented by the PaO_2_/FiO_2_ ratio), and MAP. During treatment according to Algorithm  2 all patients receive norepinephrine to maintain a MAP of ≥65 mmHg [46]. Algorithm  2 takes into account that, during later phases of treatment, in individual patients different treatment goals are necessary (negative fluid balance, positive fluid balance, or inotropic support). In addition, Algorithm  2 promotes individual treatment decisions in the light of therapeutic conflicts.

We deliberately did not include lactate measurements in our algorithms. Nevertheless, although elevated lactate is an unspecific marker of tissue hypoperfusion, serial measurements of lactate can help to assess the response of hemodynamic management strategies in patients with septic shock [22].

## 6. Hemodynamic Management in Septic Shock: Open Research Questions and Future Directions

When planning future studies on the impact of hemodynamic management on outcome in patients with septic shock various factors need to be considered.

First, although the new consensus definitions allow identifying patients with sepsis and septic shock in clinical practice, the complexity of sepsis and septic shock makes exact definitions of these “infection-related (multiple) organ dysfunction syndromes” extremely difficult. This is a problem in clinical interventional studies evaluating the effect of any therapeutic intervention in septic patients. Sepsis and septic shock are not distinct and well characterized “diseases” but rather complex syndromes that are often even accompanied by other syndromes of critical illness (such as ARDS); therefore, in clinical studies, therapeutic interventions are usually evaluated in a very heterogeneous group of septic patients [80]. This makes definite conclusions about the value of a certain intervention and the identification of distinct subgroups of septic patients who might benefit from it very challenging because possible positive or negative effects of a therapeutic approach might overlap [80, 81]. This is one reason why many randomized controlled clinical trials in septic patients fail to prove a beneficial effect of the studied intervention [80]. Therefore, future studies should precisely define the patient population studied (e.g., “patients with community-acquired septic shock of pulmonary origin” instead of “patients with sepsis or septic shock”).

In addition, complex disease syndromes require a multimodal therapeutic approach. The difficulty to prove beneficial effects of a certain single therapeutic intervention in critically ill patients has been discussed before [80]. In complex critically ill patients, it might be a basic misconception to choose mortality as the primary outcome endpoint in interventional studies. Mortality is determined by a variety of different factors thus making it very difficult to prove a clinically relevant decrease in mortality by a single intervention. Endpoints reflecting an improvement in organ dysfunction might better serve the purpose to evaluate beneficial effects of therapeutic approaches in ICU patients [80].

The complexity and heterogeneity of patients with septic shock implies individualized approaches for hemodynamic management. This includes individualized targets for hemodynamic resuscitation parameters [12, 18] and a definition of the terms “normal values” and “optimization” (in contrast to maximization). First studies on goal-directed therapy had proposed to target “supranormal” hemodynamic values in high-risk surgical patients [82]; however, this concept was later disproved in critically ill septic patients [83, 84]. In line, data from a recent study in pigs with severe acute pancreatitis as a paradigm for severe systemic infection showed that a “maximized” utilization of the cardiac preload reserve is not an “optimized” fluid management approach [85]. Normal values of hemodynamic variables show marked interindividual variability and are dependent on a variety of biometric and pathophysiologic factors [86–91]. Therefore, a “one size fits all” approach will always be deemed to fail in the hemodynamic management of critically ill patients [78]. Defining a septic patient's individual optimal values of hemodynamic variables targeted during hemodynamic management in different phases of the disease will remain a highly complex challenge but must be the focus of future research in this field.

Finally, the ultimate goal of hemodynamic management strategies based on advanced global hemodynamic variables must be to improve microcirculatory perfusion. Different technologies for the bedside assessment of the microcirculatory perfusion are available today. However, these technologies are not recommended to guide therapy outside of clinical studies [22]. The link between alterations in global hemodynamics and the microcirculation in septic shock is still not fully elucidated [12]. Therefore, therapeutic concepts for the improvement of microcirculatory perfusion are not clinically established. Future hemodynamic management concepts should therefore integrate global hemodynamic variables and variables reflecting microcirculatory perfusion.

## 7. Summary

In patients with sepsis and septic shock, the hemodynamic management in both early and later phases of these “organ dysfunction syndromes” is a key therapeutic component.

It needs however to be differentiated between EGDT as proposed for the first 6 hours of emergency department treatment by Rivers et al. in 2001 and “hemodynamic management” using advanced hemodynamic monitoring in the ICU.

Recent large trials demonstrated that nowadays protocolized EGDT does not seem to be superior to “usual care” in terms of a reduction in mortality in emergency department patients with early identified septic shock who promptly receive antibiotic therapy and fluid resuscitation.

“Hemodynamic management” comprises (a) making the diagnosis of septic shock as one differential diagnosis of circulatory shock, (b) assessing the hemodynamic status including the identification of therapeutic conflicts, and (c) guiding therapeutic interventions.

We propose two algorithms for hemodynamic management using transpulmonary thermodilution-derived variables aiming to optimize the cardiocirculatory and pulmonary status in adult ICU patients with septic shock.

The complexity and heterogeneity of patients with septic shock implies that individualized approaches for hemodynamic management are mandatory. Defining individual hemodynamic target values for patients with septic shock in different phases of the disease must be the focus of future studies.
